# *In Vivo* Electrocochleography in Hybrid Cochlear Implant Users Implicates *TMPRSS3* in Spiral Ganglion Function

**DOI:** 10.1038/s41598-018-32630-9

**Published:** 2018-09-21

**Authors:** A. Eliot Shearer, Viral D. Tejani, Carolyn J. Brown, Paul J. Abbas, Marlan R. Hansen, Bruce J. Gantz, Richard J. H. Smith

**Affiliations:** 1 Department of Otolaryngology—Head and Neck Surgery, University of Iowa Carver College of Medicine, Iowa City, Iowa USA; 2 Interdepartmental PhD Program in Genetics, University of Iowa, Iowa City, Iowa USA; 3 Department of Molecular Physiology & Biophysics, University of Iowa College of Medicine, Iowa City, Iowa USA

## Abstract

Cochlear implantation, a surgical method to bypass cochlear hair cells and directly stimulate the spiral ganglion, is the standard treatment for severe-to-profound hearing loss. Changes in cochlear implant electrode array design and surgical approach now allow for preservation of acoustic hearing in the implanted ear. Electrocochleography (ECochG) was performed in eight hearing preservation subjects to assess hair cell and neural function and elucidate underlying genetic hearing loss. Three subjects had pathogenic variants in *TMPRSS3* and five had pathogenic variants in genes known to affect the cochlear sensory partition. The mechanism by which variants in *TMPRSS3* cause genetic hearing loss is unknown. We used a 500-Hz tone burst to record ECochG responses from an intracochlear electrode. Responses consist of a cochlear microphonic (hair cell) and an auditory nerve neurophonic. Cochlear microphonics did not differ between groups. Auditory nerve neurophonics were smaller, on average, in subjects with *TMPRSS3* deafness. Results of this proof-of-concept study provide evidence that pathogenic variants in *TMPRSS3* may impact function of the spiral ganglion. While ECochG as a clinical and research tool has been around for decades, this study illustrates a new application of ECochG in the study of genetic hearing and deafness *in vivo*.

## Introduction

Hearing loss is the most common sensory deficit in humans. Our understanding of its biology and molecular physiology has been guided in large part by the discovery of genes implicated in both syndromic (several hundred genes) and non-syndromic (over 100 genes) forms of hearing loss (http://hereditaryhearingloss.org). Pathogenic variants in the *TMPRSS3* gene cause autosomal recessive non-syndromic hearing loss (ARNSHL) at the DFNB8 and DFNB10 loci^1^, two loci mapped over 20 years ago in consanguineous families with disparate phenotypes. In the DFNB10 family, the deafness was congenital and severe-to-profound while in the DFNB8 family, it was post-lingual and progressive^2,3^. Both families were ultimately found to segregate pathogenic variants in *TMPRSS3*. The phenotypic differences are consistent with more recent data that show clear correlations with *TMPRSS3* variant type and the severity and onset of associated hearing loss^4–7^.

*TMPRSS3* encodes transmembrane serine protease 3, the function of which is unknown. Four studies show expression of *TMPRSS3* in inner and outer cochlear hair cells as well as spiral ganglion neuronal bodies by *in situ* hybridization^8,9^, RT-PCR and immunohistochemistry^10^, and immunofluorescence and immunohistochemistry^11^; one study shows robust expression in the sensory components of the vestibular system in mice^9^. Expression in both the spiral ganglion and the cochlear sensory organ indicates a possible functional role in both tissues; however, *in vitro* studies have failed to clarify the mechanism by which *TMPRSS3* causes deafness. A yeast-based assay has shown that pathogenic mutations in *TMPRSS3* negatively affect proteolytic activity of the enzyme and effect of mutations on proteolytic activity could explain the variable phenotype of *TMPRSS3* deafness^12^. Other early *in vitro* studies in the Xenopus oocyte expression system demonstrated the existence of auto-catalytic processing by which *TMPRSS3* becomes active and that a specific sodium channel (ENaC) could be a substrate of *TMPRSS3*^8^. However, this channel is not expressed in human cochlear hair cells. More recent data support a role in *TMPRSS3* function in localization of the potassium channel Kcnma1, which is critical for maintenance of the resting potential of cochlear inner hair cells and for their survival at the onset of hearing^13^. Data also show that the function of *TMPRSS3* in spiral ganglion neurons is critical for their survival, as demonstrated by Li *et al*. using siRNA and miR-specific gene suppression^14^.

Recently we showed that deleterious variants in *TMPRSS3* are the most common genetic cause of hearing loss in post-lingually deafened adult cochlear implant (CI) users^15^. In the same study we also showed that as a group, CI users with deleterious genetic variants in genes expressed in the spiral ganglion or auditory nerve have poorer overall outcomes as compared to other CI cohorts. These observations are potentially important as cochlear implantation has become the standard surgical treatment for persons with severe-to-profound hearing loss. More than 219,000 persons have undergone cochlear implantation since 1985 when the technology was first approved by the FDA. Hybrid cochlear implants were developed more recently as an option for individuals with high frequency hearing loss who do not benefit from conventional hearing aids but are not qualified for traditional cochlear implantations. Hybrid CI users hear high frequencies via electrical stimulation but continue to use acoustic stimulation to hear low frequencies^16^. This technology has resulted in significant an expansion of CI candidacy.

It has long been possible to use an intracochlear cochlear implant electrode to record *electrically* evoked neural responses arising from the auditory nerve of CI users^17^. In addition, electrocochleography (ECochG) has been used in clinical and research practice for decades to measure cochlear hair cell and neural responses from normal hearing and hearing impaired populations in response to *acoustic* stimulation. The ability to record such responses requires that patients have residual acoustic hearing, precluding applications in CI populations. ECochG recordings have four different and overlapping components: the cochlear microphonic (CM) and the summating potential (SP), generated from cochlear hair cells^17–19^; and two neural responses, the compound action potential (CAP) and auditory nerve neurophonic (ANN)^20–22^.

ECochG has received renewed attention in recent years due to potential clinical applications in CI populations, such as predicting post-operative speech outcomes^23,24^, monitoring insertion trauma from cochlear implantation^25,26^, and monitoring changes in acoustic hearing over time in patients with preserved acoustic hearing in the implanted ear^27,28^. Here we propose a novel application of ECochG to study the functional effects of pathogenic variants in *TMPRSS3* in humans. We hypothesized, based on our previous work, that these individuals may exhibit abnormal function at the level of the spiral ganglion or auditory nerve^15^. Results show that, on average, when compared to results obtained from five Hybrid CI subjects carrying pathogenic variants in genes affecting the cochlear sensory partition (Sensory Group), the three Hybrid cochlear implant users with pathogenic variants in *TMPRSS3* have ECochG responses that are consistent with a significant reduction in auditory nerve/spiral ganglion function. Results of this study, while seriously underpowered, provide evidence that pathogenic variants in *TMPRSS3* could help us refine our understanding of the molecular physiology of hearing and deafness.

## Results

Table 1 describes the eight subjects who participated in this study. All eight subjects were implanted at the University of Iowa Hospitals and Clinics with a Nucleus Hybrid CI, had retained acoustic hearing following surgery, and had a positive genetic test result. Three subjects had pathogenic genetic variants in *TMPRSS3* causing DFNB8 deafness (*TMPRSS3* Group). Five subjects had pathogenic genetic variants in genes known to affect the cochlear sensory organ including *KCNQ4* (required for outer hair cell function), *TMC1* (required for mechanotransduction of inner hair cells), *MYO6*, *MYO15A*, and *LOXHD1* (all required for inner and outer hair cell development). These five subjects formed the comparison Sensory Group.

**Table 1:** Subject Characteristics, Results of Genetic Testing and Speech Perception Outcomes.

Group	Subject	Implant	Age at implant (yrs)	Implant use at ECochG testing (mo.)	500 Hz threshold (dB HL)	Electric alone CNC Score (%)	Acoustic + Electric CNC Score (%)	Causative Gene	Causative Mutation	Inheritance	Deafness locus
SENSORY	S1	L24	55	48	50	44	73	*TMC1*	p.Ser208Arg c.1204_1208	Dominant	DFNA36
	S2	L24	49	12	80	72	94	*KCNQ4*	delGCGCC c.1134delC	Dominant	DFNA2
	S3	L24	18	27	80	72	81	*MYO15A*	c.6482delC p.Arg1204Glnp.	Recessive	DFNB3
	S4	L24	12	35	90	68	74	*MYO6*	His246Arg p.Leu635Pro	Recessive	DFNB37
	*S5	L24	24	1	*70	35	*35	*LOXHD1*	p.Arg266Gln p.Ala138Glu	Recessive	DFNB77
*TMPRSS3*	**T1	L24	64	22	**NR	2	**2	*TMPRSS3*	c.208delC p.Ala425Thr	Recessive	DFNB8
	T2	S8	53	103	65	29	70	*TMPRSS3*	p.Ala138Glu p.Ala425Thr	Recessive	DFNB8
	T3	L24	38	12	55	83	94	*TMPRSS3*	c.1345-2A > G	Recessive	DFNB8

*Subject S5 underwent ECochG testing at 1 month post device activation. Speech perception was not measured at that time. She lost all residual acoustic hearing by 3 months post activation. Speech perception scores listed in this table were measured using electric alone stimulation and presumed to be the same for acoustic and electric stimulation because her hearing loss was so substantial.

**Subject T1 had residual acoustic hearing at 125 Hz and 250 Hz but did not have measurable hearing at 500 Hz. He also did not use the acoustic component of his implant. Here we assume his acoustic + electric CNC score is the same as his electric-alone score.

**Figure 1:**
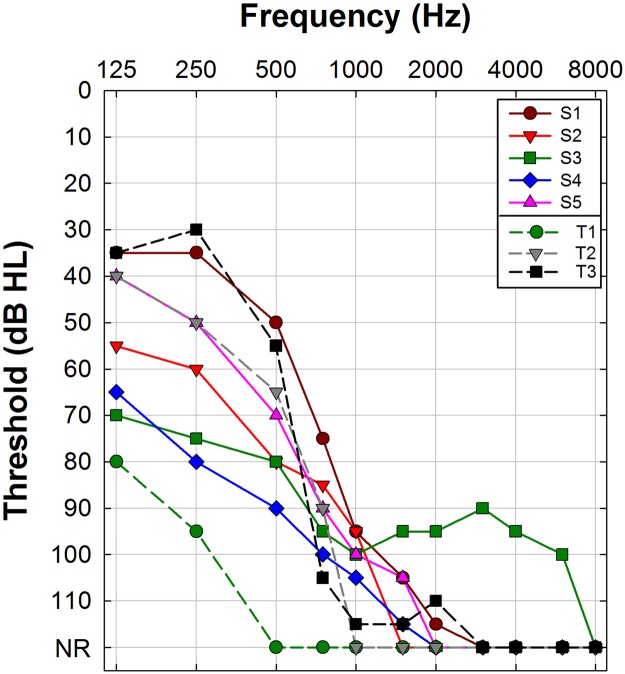
Post-operative audiograms in the implanted ear of the 8 subjects. Audiograms for subjects in the Sensory group are shown with symbols connected by solid lines (S1–S5) and that of the *TMPRSS3* group with symbols connected by dashed lines (T1–T3). NR indicates no response at limits of the audiometer.

Post-operative audiometric thresholds are shown in Fig. 1 and were obtained on the same day of ECochG testing. Speech perception outcomes (Table 1) were obtained from chart review and were collected either on the same day or at a previous visit (see methods section). Subject S1 was seen for evoked potential testing at 1 month post activation. No speech perception data was collected at the time of ECochG testing. She experienced complete loss of residual acoustic hearing a few weeks later. Subject T1 had limited residual acoustic hearing precluding use of the acoustic (hearing aid) component of the implant system. For both subjects, we report speech perception scores measured in the electric-only stimulation mode and use that same score to estimate performance in the acoustic + electric stimulation mode to indicate no benefit from acoustic stimulation. The average speech perception score (±1 sd) in the Sensory Group was 58.2 ± 17.4% in the electric-only listening mode and improved to 71.4 ± 22% with combined acoustic and electrical stimulation. The average speech perception score for the *TMPRSS3* Group (±1 s.d.) was 38 ± 41.2% in the electric-only stimulation mode and improved to 55.3 ± 47.7% in the combined acoustic and electric stimulation mode. A two-way repeated measures ANOVA with group (Sensory vs *TMPRSS3*) as the between-subjects factor and listening mode (electric-only vs acoustic + electric) as the within-subjects factor showed a significant main effect of listening mode (F_1,6_ = 7.128, p < 0.05). The effect of subject group (Sensory vs *TMPRSS3*) was not statistically significant (p > 0.05). No significant interaction (F_1,6_ = 0.131, p > 0.05) was found for this small sample of subjects.

**Figure 2:**
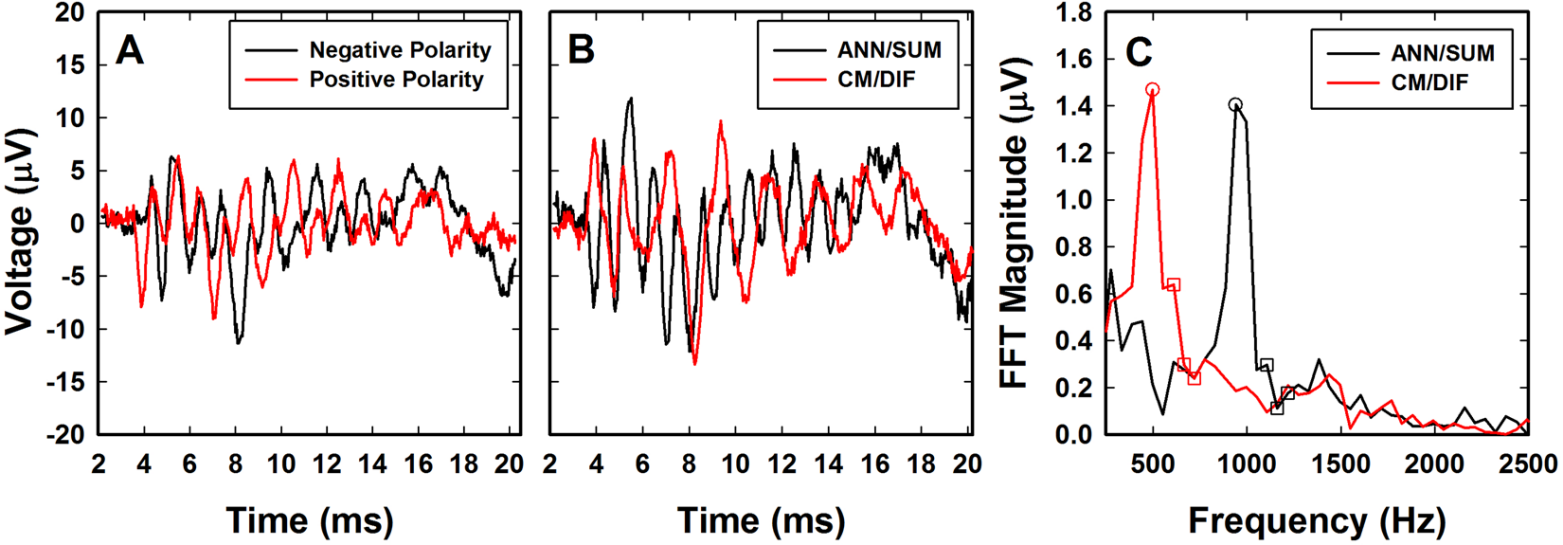
Electrophysiological results for subject S5 demonstrating ANN/SUM and CM/DIF components using *in vivo* electrocochleography. (**A**) Waveforms recorded using a 500 Hz tone burst presented in both stimulus polarities at 105 dB SPL for subject S5. (**B**) The waveforms in (**A)** were combined to form ANN/SUM and CM/DIF waveforms. (**C**) ANN/SUM and CM/DIF waveforms shown in (**B**) were analyzed using Fast Fourier Transform (FFT). The open circles indicate the FFT component used to quantify magnitude of the ANN/SUM and CM/DIF responses. The open squares indicate the three points used to estimate noise floor for this individual (see text).

Figure 2 shows electrophysiologic recordings obtained from an individual study participant (S5) to illustrate measurement procedures. Two opposite polarity, 500 Hz tone bursts were presented. The waveforms shown in Fig. 2A exhibit clear periodicity and reverse polarity when the stimulus polarity is reversed. We assume that these recordings include contributions both from cochlear hair cells and the auditory nerve. Figure 2B shows the waveforms that result when the two responses in Fig. 2A are subtracted and added together. To the extent that the CM is linear and follows the stimulus waveform, subtracting the two recordings shown in Fig. 2A will enhance the contribution of the cochlear hair cells and minimize contributions from the auditory nerve. We refer to the subtracted waveform shown in Fig. 2B as the CM/DIF potential. Adding the two waveforms shown in Fig. 2A minimizes contribution from the hair cells (CM) and helps isolate the response from the auditory nerve. This derived response is also shown in Fig. 2B and will include a combination of the auditory nerve neurophonic (ANN) and compound action potential^29–31^. The ANN will be a periodic potential with significant energy at twice the stimulus frequency (1000 Hz in this case). We note that neither manipulation results in complete separation of the CM and ANN response^32^. Figure 2C shows the results of a Fast Fourier Transform (FFT) of the CM/DIF and ANN/SUM potentials. The FFT of the CM/DIF response has a strong 500 Hz component and the ANN/SUM FFT has a strong 1000 Hz component. The noise floor was estimated by computing the average of the FFT for three bins between 553 and 719 Hz for the CM/DIF responses and between 1106 and 1217 Hz for the ANN/SUM response.

**Figure 3:**
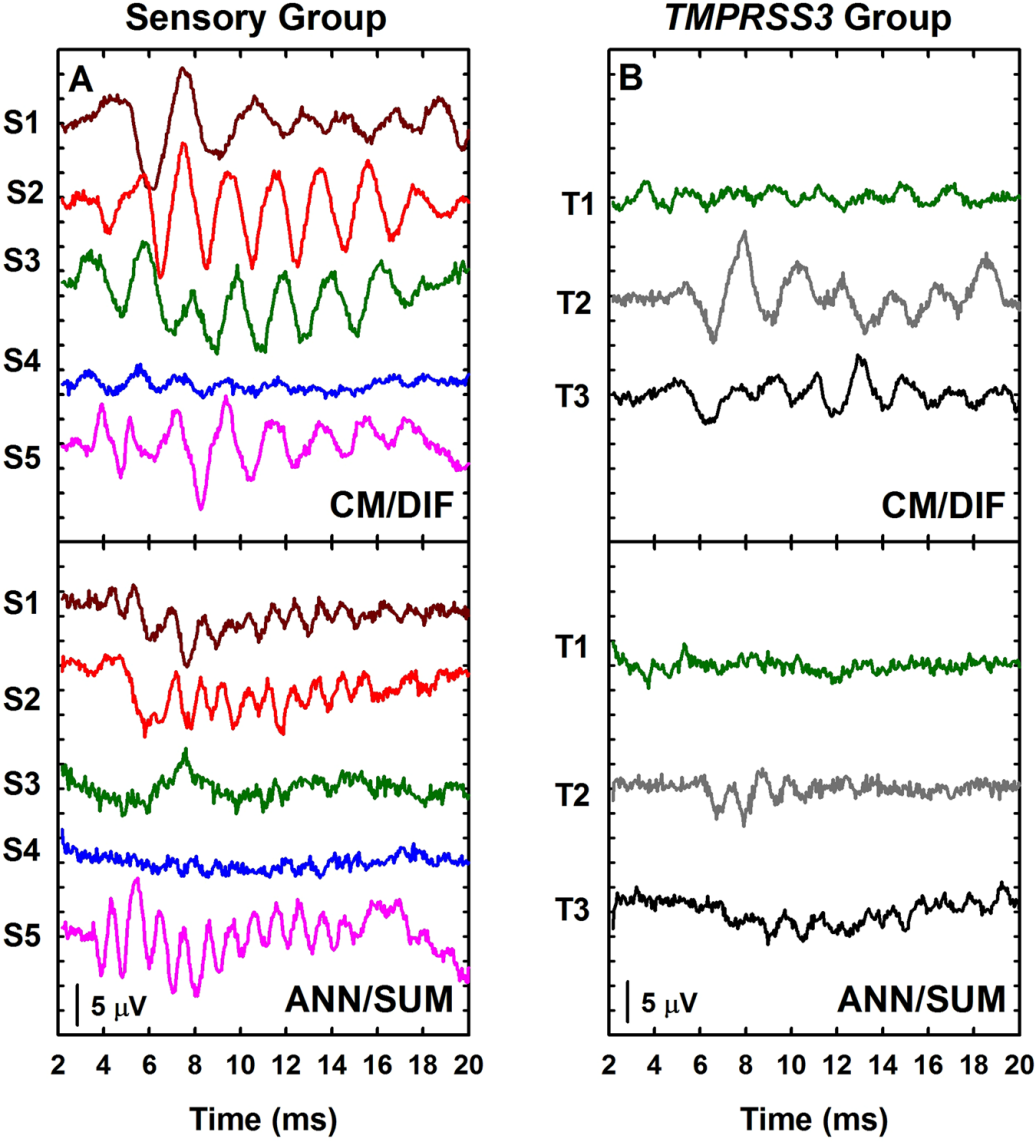
Electrophysiological summaries for all subjects in both groups. CM/DIF and ANN/SUM responses were recorded from individuals in both the Sensory and *TMPRSS3* Groups.

Figure 3 shows CM/DIF and ANN/SUM waveforms recorded for all eight subjects. We ensured that all responses were free from artifact by performing a control recording where the stimuli were presented but the insert earphone removed. These recordings were free of any physiologic responses. Clearly there is significant variation in the response waveforms both across subjects and across time within a subject. This variation makes it difficult to quantify the overall strength of the response from the waveform directly. Instead, we analyze these recording more objectively in the frequency domain.

**Figure 4:**
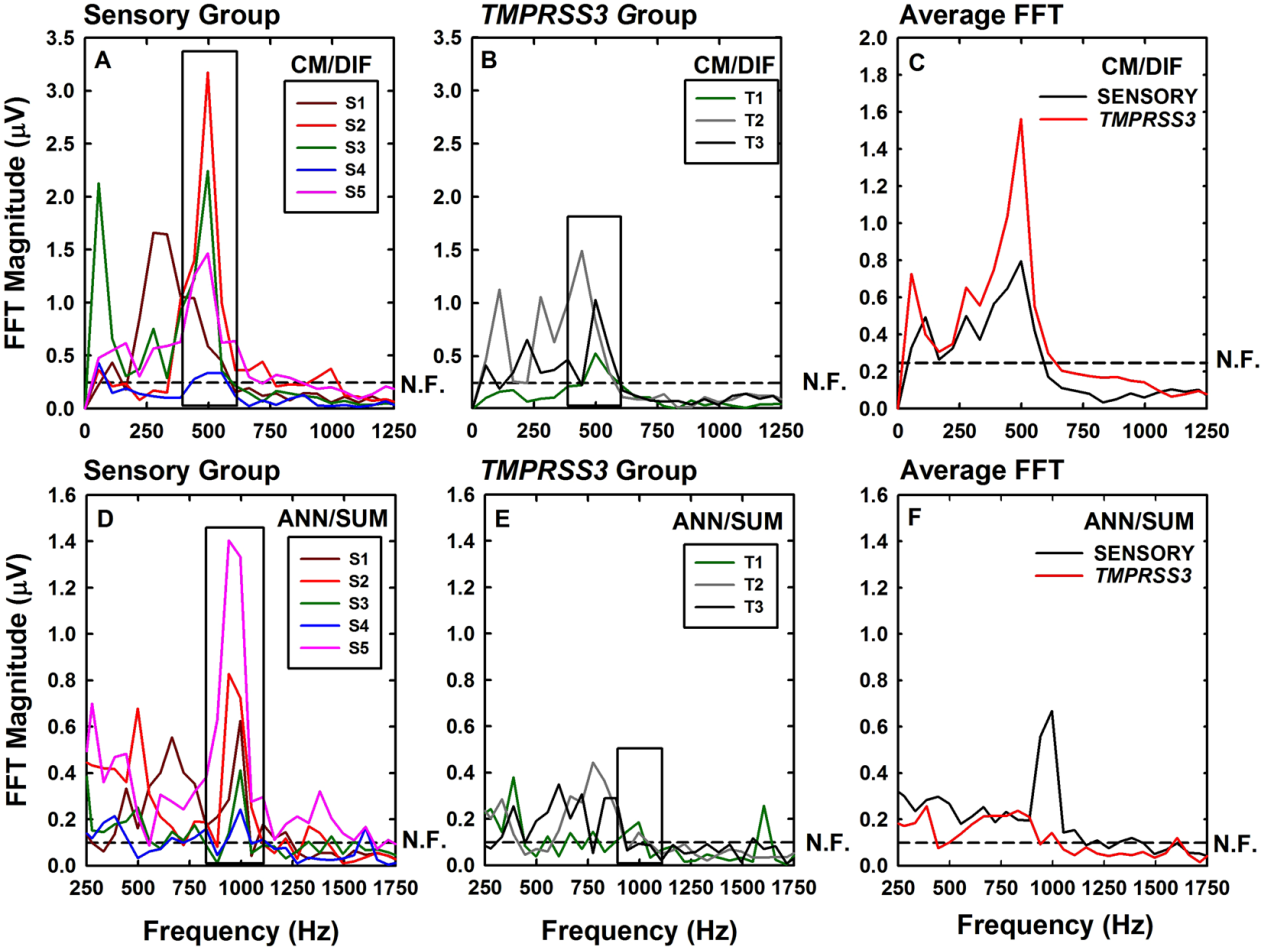
Electrophysiological analysis comparison for all subjects using FFT (Fast Fourier Transform). The waveforms from Fig. 3 were analyzed using a FFT. The upper row (Panels A–C) shows the results of frequency analysis of the CM/DIF responses; the lower row (Panels D–F) shows FFTs computed using the ANN/SUM responses. The two panels (A,D) on the left show results from the Sensory group. The two panels in the center (**B**,**E**) show results from the *TMPRSS3* group. The two panels on the right (**C**,**F**) show the average FFT for the subjects in both groups. The dashed lines indicate the estimate of the noise floor for all eight subjects. The boxes indicate the frequency region where we expect to find a measurable ANN/SUM or CM/DIF response.

Figure 4 shows the FFT analysis for individual subjects in both groups. Average FFT recordings are also shown as a way to help illustrate trends. Subjects in both groups have 500 Hz components that are above the noise floor, suggesting that all eight subjects have viable cochlear hair cells. We expect subjects in both groups to have viable hair cells because they all have residual acoustic hearing. While smaller in amplitude, all five subjects in the Sensory Group also have identifiable peaks at 1000 Hz in the FFT of the ANN/SUM responses that are above the noise floor of the measurement system. In contrast, the 1000 Hz component of the ANN/SUM responses for the three subjects in the *TMPRSS3* group are small and only one is above the noise floor of the measurement system. To the extent that the ANN/SUM responses reflects neural activity, these results are consistent with a neural (spiral ganglion) site of lesion for subjects in the *TMPRSS3* Group.

**Figure 5:**
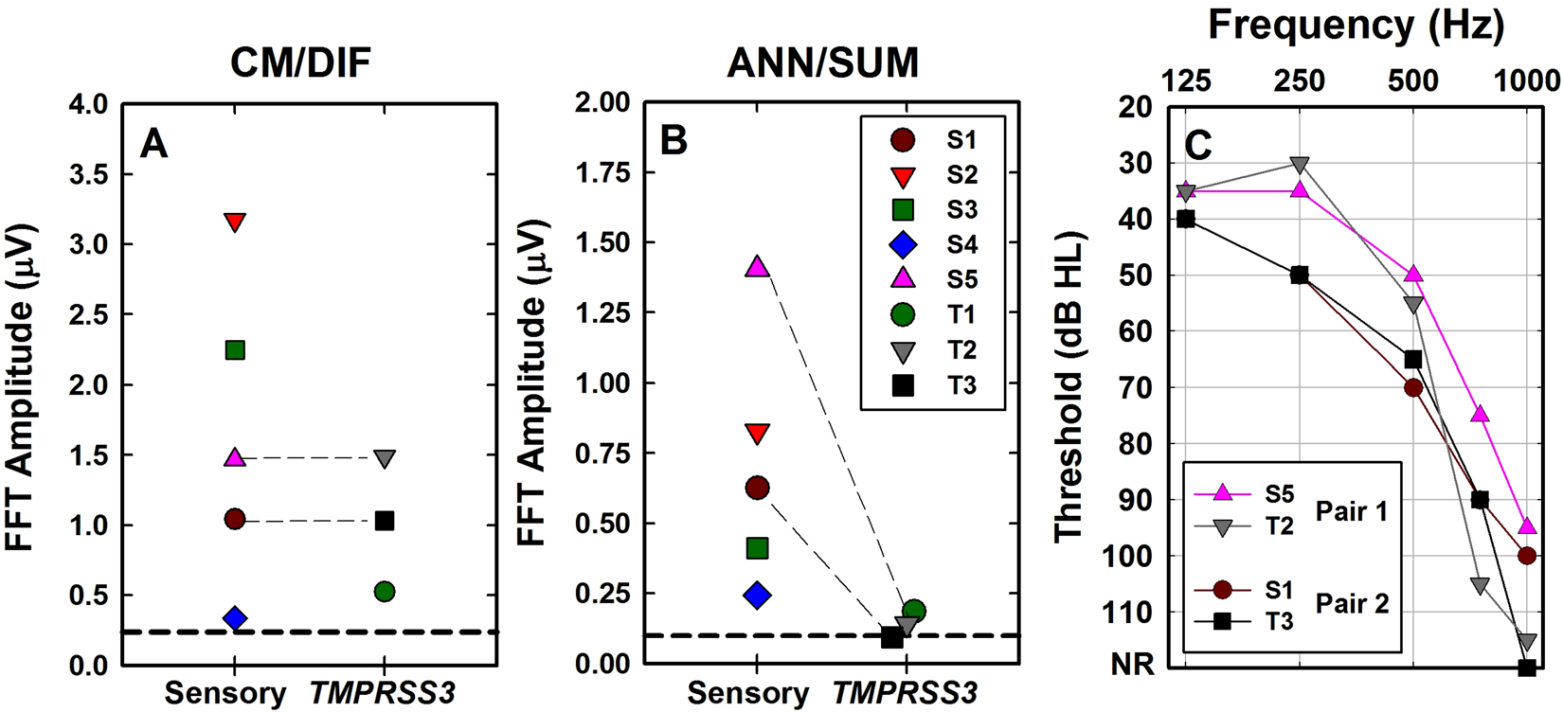
Summary of FFT results by group and comparisons of two matched pairs of subjects. Panels A and B show the 500 Hz component and the 1000 Hz component of the FFT amplitude, respectively, for both subject groups. In both panels, dashed lines connect results from individuals in the two groups who have similar audiograms (Panel C).

Figure 5A,B shows the magnitude of the 500 Hz component of the CM/DIF responses and the 1000 Hz component of the ANN/SUM responses for subjects in both groups. There is considerable across- and within-group variance in response magnitudes for both subject groups, which may reflect the variance in audiometric threshold in both groups. While significant correlations between audiometric thresholds and CM/ANN thresholds have previously been reported for a large cohort of subjects^27,28^, it is not known whether audiometric thresholds correlate well with suprathreshold CM/ANN amplitudes. We note that subjects in the *TMPRSS3* group had the smallest ANN/SUM responses, consistent with a neural/spiral ganglion site of lesion. However, we knowledge that the variance in audiometric thresholds may affect ANN/SUM responses, which led to an alternative analysis shown in Fig. 5C. Here, we show audiometric thresholds for two pairs of subjects (S5 and T2, S1 and T3). The subjects in each pair have almost identical audiograms but one subject in each pair is from the sensory group and the other from *TMPRSS3*. Note that for both subject pairs, the CM/DIF responses were almost identical (see individual data points connected by dashed lines in Fig. 5A). However, the subjects in each pair from the *TMPRSS3* Group (T2 and T3) had ANN/SUM responses that were in or very close to the noise floor of the measurement system. These differences are most likely not attributable to differences in audiometric thresholds.

## Discussion

In this study, we present a novel *in vivo* human electrophysiological application to assess the functional impact of a genetic lesion on the human peripheral auditory system. ECochG has had widespread applications in clinical practice and research studies for several decades^33^. It has typically been evoked using acoustic stimulation recorded via an electrode placed in the ear canal, on the tympanic membrane or occasionally on the promontory of the middle ear. ECochG amplitudes increase as the position of the recording electrode is moved closer toward the cochlea^22,34^. In this study, we recorded the ECochG using an intracochlear electrode, providing the most response possible without resorting to invasive methods. While the technique is not new, the application itself is. To our knowledge, this is the first study where intracochlear measures of hair cell/neural responses from the auditory periphery have been used to assess the functional impact of a genetic lesion on the human peripheral auditory system.

ECochG recordings have four different and overlapping components: the cochlear microphonic (CM) and the summating potential (SP), generated from cochlear hair cells^17–19^; and two neural responses, the compound action potential (CAP) and auditory nerve neurophonic (ANN)^20–22^. For decades, clinicians have recorded responses using opposite polarity stimuli, adding them to minimize the CM or subtracting them to emphasize responses recorded from the auditory nerve. The addition/subtraction technique we have used to segregate the response of hair cells from the auditory nerve was also used in these earlier studies. The extent to which the two responses can be isolated has been a matter of debate^29–32^. However, we argue that while the method we use to separate the hair cell response from the neural response may not be perfect, it provides the most direct measure of the response of the peripheral auditory system to acoustic stimulation that is available to date. This study is novel because, for the first time, we attempt to validate the method for assessing individuals with a known genetic diagnosis.

Other investigators have recorded this response from cochlear implant users by placing an extracochlear electrode on the round window at the time of cochlear implant surgery. These investigators have reported significant correlations to post implant speech perception in quiet^23,24^. Correlations between CM/DIF and ANN/SUM thresholds and audiometric thresholds have also been reported by our group and another group^27,28^. There are also research teams who are working on developing methods to use intracochlear recordings to monitor cochlear function and guide insertion of the intracochlear electrode array^25,33,35^. This study, though, is the first to use this electrophysiological measure of the peripheral auditory system to study Hybrid cochlear implant users who carry a genetic diagnosis.

We expected that that all eight subjects would have measurable cochlear microphonics since all eight had residual acoustic hearing in the implanted ear. We hypothesized that the pathogenic genetic variants in *TMPRSS3* would impact spiral ganglion neurons specifically and that we could measure this impact by analyzing neural (ANN/SUM) responses. We anticipated that subjects in the *TMPRSS3* group would have smaller neural responses (ANN/SUM) than subjects in the sensory group. While our results are preliminary in nature, they are consistent with that hypothesis (see Figs 4–5). That trend is also apparent for subjects with identical audiograms (see Fig. 5). We are excited by this finding because it illustrates a new role for intracochlear ECochG recordings. That is, refining our understanding of the pathophysiology of genetic hearing loss and/or deafness.

Previously we showed that on speech recognition metrics, CI recipients with pathogenic genetic variants known to affect the sensory structures of the ear (e.g. inner or outer hair cells) outperform subjects with pathogenic genetic variants in genes expressed in neural structures (e.g. spiral ganglion cells)^15^. That observation suggests that in the current study, individuals in the sensory group would have better speech perception scores than the individuals in the *TMPRSS3* group. This was not the case for the eight subjects included in this study. There was considerable overlap and no statistically significant differences between the two groups on speech perception measures and audiometric thresholds (see Table 1 and Fig. 1). This may reflect the small sample size. Indeed, a review of every published subject with *TMPRSS3* mutations and a CI (n = 27), showed variable results in regards to outcomes and a lack of uniform outcome measures for comparison (see Supplemental Table 1). Other reports also suggest that speech perception and audiometric thresholds can vary widely for individuals with a diagnosis that implicates the auditory nerve (e.g. auditory neuropathy or vestibular schwannomas^36–38^. These observations underscore that audiometric thresholds and speech performance metrics do not completely reflect underlying peripheral deficits and that ECochG may assist in understanding the pathophysiology of genetic hearing loss.

One may presume that electrophysiological measures of neural function may reflect speech perception outcomes; however, the literature suggests mixed findings. Speech perception outcomes are not necessarily correlated with electrically evoked neural responses^39–42^ and post-mortem spiral ganglion neuron counts^43–46^. Only recently has it been suggested that acoustically evoked potentials (ECochG) could correlate with CI speech perception outcomes^23,24,47^. Mixed findings are not surprising as all these evoked potentials reflect a peripheral response while speech perception requires peripheral, central, and cognitive processes. Both peripheral and central measures may be needed to increase the predictive power of electrophysiologic measures^48^.

An important limitation of this work is the small number of subjects, which limits meaningful statistical analysis. As such, our results should be considered proof-of-concept only. Another weakness of this study, as we alluded to previously, is that the method used to isolate hair cell and neural contributions to an acoustic stimulus are not clean. While animal data suggests that the ANN/SUM responses arise from auditory neurons^30,31^, they may also reflect distortion of the hair cell transduction process^32^. It seems likely that responses recorded from a normal-hearing animal model will not be identical to responses recorded from an impaired human cochlea. While we cannot argue that we have completely separated hair cell responses from auditory nerve responses, we have argued in our previous study that the derived CM/DIF and ANN/SUM responses are at least biased toward the cochlear hair cells and auditory nerve, respectively^27^.

These limitations notwithstanding, we are encouraged by our results. These *in vivo* recordings, obtained using a nearfield electrode, provide the most direct measure of the response of the peripheral auditory system to acoustic stimulation available to date. While similar recordings could be obtained using surface electrodes, these potentials are much smaller^22,34^. Intracochlear ECochG provides a level of specificity about the site-of-lesion in the peripheral auditory system that is not available from behavioral testing. Future studies examining other genetic causes and etiologies of hearing loss are planned and we hope will provide valuable insight into the molecular physiology of hearing and deafness.

## Methods

### Subjects

Forty-four subjects received a Nucleus Hybrid CI at the University of Iowa Hospitals and Clinics between 2001 and 2016 and underwent comprehensive genetic testing (see below); eight had a confirmed genetic diagnosis and participated in this study (Table 1). Each participant met standard pre-operative criteria for implantation with a Nucleus Hybrid cochlear implant and were implanted by authors MRH or BJG. Seven received the L24 electrode array. The Nucleus L24 electrode array was approved by the Food and Drug Association (FDA) in March 2014. It has 22 electrodes mounted on a 16 mm Silastic carrier, is inserted 16–17 mm into the cochlea, and spans approximately 270° of the basal turn of the cochlea^49–51^. One subject (T2) received an S8 electrode array as part of an FDA-approved, investigational trial. This electrode array features six electrodes mounted on a Silastic carrier that is 10 mm in length and, when inserted fully, spans 190–200^o^ of the basal turn of the cochlea^52,53^. This study was approved by the Institutional Review Board of the University of Iowa and all research was performed in accordance with University of Iowa guidelines on Human Subjects Research. All subjects signed an informed consent document.

### Genetic testing

Comprehensive genetic testing was performed with the OtoSCOPE® platform using DNA extracted from peripheral blood according to standard practices (see https://www.medicine.uiowa.edu/morl/otoscopegenes/ for complete gene list)^54,55^. Genetic testing results were discussed at a multidisciplinary meeting with geneticists, bioinfomaticians, graduate students, auditory research scientists and otolaryngologists to determine the likely genetic cause of deafness, if any, for each individual. OtoSCOPE v6 or v7 was used, with the primary difference being the addition of newly discovered deafness genes in v7.

Three subjects (T1–T3) had pathogenic variants in *TMPRSS3* (see Table 1) and were diagnosed with DFNB8-related hearing loss. T1–T3 ranged in age from 38 to 64 years (mean = 51.7 yrs) at the time of implantation. We refer to these subjects as the *TMPRSS3* Group.

Five subjects (S1–S5) had pathogenic variants in *TMC1*, *KNCQ4*, *MYO15A*, *MYO6* and *LOXHD1*, genes expressed in the sensory portion of the cochlea (see, Table 1). Two subjects reported congenital onset of hearing loss, two reported childhood-onset hearing loss, and one reported adult-onset hearing loss. Implantation with a Nucleus Hybrid device was done between 12 and 55 years of age (mean = 31.6 yrs). We refer to these subjects as the Sensory Group.

### Audiometric testing

Audiometric thresholds were obtained using a modified Hughson-Westlake procedure^56^ at the time of ECochG testing (Fig. 1). Performance on the monosyllabic CNC word test^57^ was obtained from a review of clinical records and is shown on Table 1. This test consists of a paired list of 25 words each, presented in quiet at 60 dB SPL from a loudspeaker 1 meter away from the subject at 0 degrees azimuth. Subjects repeat the words they hear and their responses are scored in percent correct. Each subject was tested twice–once using both the electric and acoustic components of the Hybrid CI and once using only the electrical component. The non-implanted ear was plugged and muffed during speech testing. Speech perception testing is not done routinely in our clinic until three months post implant. The test results shown in Table 1 were recorded at the time of ECochG testing. However, in two cases (subjects S1 and S5), the test times vastly differed. For S1, ECochG testing was done at 48 months post-implant activation, but we used her 12 month speech perception scores since that dataset contained scores in both electric-only and acoustic + electric stimulation modes. Appointments after 12 months demonstrated stability in speech scores but did not contain both stimulation modes at every appointment. For subject S5, her ECochG testing was done at 1 month. Speech perception testing is not usually done until 3 month post-activation, per our clinical protocol. For S5, she lost all of her residual acoustic hearing before her 3 month visit. We used her 22 month speech perception score, though her score at 12 month was also similar.

Additionally, since subjects S5 and T1 had limited to no residual acoustic hearing, they did not use the acoustic component of their Hybrid CI system. For both subjects, speech testing was conducted in the electric-only stimulation mode. Scores listed under the acoustic plus electric mode in Table 1 were assumed to be the same as electric-only, to reflect lack of acoustic benefit.

### Electrophysiological testing

ECochG recordings were obtained using Custom Sound EP software (ver 3.2) and a research patch provided by Cochlear Ltd, which allowed us to trigger a signal generator. The acoustic stimulus was a 500 Hz tone burst that was 12 ms long and was presented in both positive- and negative-leading polarities to the implanted ear via an ER-3A insert earphone at a rate of 10 Hz. Tonebursts were presented at the highest level that was still comfortable for the subject. Stimulation levels ranged from 100–110 dB peSPL. ECochG responses were recorded from the most apical electrode in the array. Each ECochG recording was an average of 200 stimulus presentations. Responses were analyzed in the frequency domain. More details regarding the recording and analysis procedures are provided in our previous study^27^.

## Electronic supplementary material

Supplementary Information
