# Supplementary material for: In Vivo Electrocochleography in Hybrid Cochlear Implant Users Implicates TMPRSS3 in Spiral Ganglion Function
Source: Sci Rep. 2018 Sep 21;8:14165. doi: 10.1038/s41598-018-32630-9 (PMC6154996; doi:10.1038/s41598-018-32630-9)
Supplement: Supplementary file 1 — Supplementary Information [file 41598_2018_32630_MOESM1_ESM.docx]

# Supplemental Information

***In Vivo* Electrocochleography in Hybrid Cochlear Implant Users Implicates *TMPRSS3* in Spiral Ganglion Function**

## A. Eliot Shearer, Viral D Tejani, Carolyn J Brown, Paul J Abbas,

## Marlan R Hansen, Bruce J Gantz, Richard JH Smith

Supplemental Table 1. Review of manuscripts presenting data on subjects with deafness-causing *TMPRSS3* mutations and cochlear implant outcome. Outcomes are mixed and there is a lack of uniformity on performance outcomes that makes pooled analysis difficult.

| Manuscript | N | CI Outcome Result for *TMPRSS3* subjects |
| --- | --- | --- |
| Weegerink et al 2011 | 8 | Better performance than reference group, word recognition score average of 64.6% for five subjects with data |
| Eppsteiner et al 2012 | 2 | Poor performance, average word recognition score of 30% |
| Chung et al 2014 | 2 | Mean open set sentence score was the same as a control group |
| Elbracht et al 2014 | 2 | “Good” performance but no detailed data presented |
| Miyawaga et al 2015 | 3 | “Good” performance but no detailed data presented |
| Battelino et al 2015 | 4 | “Good” performance but no detailed data presented |
| Gao et al 2017 | 1 | “Good” performance but no detailed data presented |
| Shearer et al 2017 | 5 | Statistically lower performance than control groups |
